# Applied machine learning in intelligent systems: knowledge graph-enhanced ophthalmic contrastive learning with “clinical profile” prompts

**DOI:** 10.3389/frai.2025.1527010

**Published:** 2025-03-12

**Authors:** Mini Han Wang, Jiazheng Cui, Simon Ming-Yuen Lee, Zhiyuan Lin, Peijin Zeng, Xinyue Li, Haoyang Liu, Yunxiao Liu, Yang Xu, Yapeng Wang, José Lopes Camilo Da Costa Alves, Guanghui Hou, Junbin Fang, Xiangrong Yu, Kelvin Kam-Lung Chong, Yi Pan

**Affiliations:** ^1^Zhuhai Precision Medical Center, Zhuhai People's Hospital, The Affiliated Hospital of Beijing Institute of Technology, Zhuhai Clinical Medical College of Jinan University, Zhuhai, China; ^2^Department of Ophthalmology and Visual Sciences, Faculty of Medicine, The Chinese University of Hong Kong, Shatin, Hong Kong SAR, China; ^3^Zhuhai Institute of Advanced Technology, Chinese Academy of Sciences (CAS), Zhuhai, China; ^4^Beijing Normal University - Hong Kong Baptist University United International College, Zhuhai, China; ^5^Department of Food Science and Nutrition, Hong Kong Polytechnic University, Kowloon, Hong Kong SAR, China; ^6^Perspective Technology Group, Zhuhai, China; ^7^Beijing Institute of Technology, Zhuhai, China; ^8^Department of Ophthalmology, Tianjin Medical University, Tianjin, China; ^9^Faculty of Applied Sciences, Macao Polytechnic University, Macao, Macao SAR, China; ^10^Digital Healthcare and Artificial Intelligence Association, Macao, Macao SAR, China; ^11^Faculty of Business, City University of Macau, Macao, Macao SAR, China; ^12^Zhuhai Aier Eye Hospital, Zhuhai, China; ^13^College of Science & Engineering, Jinan University, Shenzhen, China; ^14^Zhuhai People's Hospital (Zhuhai Clinical Medical College of Jinan University), Zhuhai, China; ^15^Shenzhen Key Laboratory of Intelligent Bioinformatics, Shenzhen Institute of Advanced Technology, Shenzhen, China

**Keywords:** machine learning, medical intelligent systems, ophthalmic disease detection, knowledge graph, contrastive learning, clinical profile prompts, interpretable artificial intelligence

## Abstract

**Introduction:**

The integration of artificial intelligence (AI) into ophthalmic diagnostics has the potential to significantly enhance diagnostic accuracy and interpretability, thereby supporting clinical decision-making. However, a major challenge in AI-driven medical applications is the lack of transparency, which limits clinicians’ trust in automated recommendations. This study investigates the application of machine learning techniques by integrating knowledge graphs with contrastive learning and utilizing “clinical profile” prompts to refine the performance of the ophthalmology-specific large language model, MeEYE, which is built on the CHATGLM3-6B architecture. This approach aims to improve the model’s ability to capture clinically relevant features while enhancing both the accuracy and explainability of diagnostic predictions.

**Methods:**

This study employs a novel methodological framework that incorporates domain-specific knowledge through knowledge graphs and enhances feature representation using contrastive learning. The MeEYE model is fine-tuned with structured clinical knowledge, enabling it to better distinguish subtle yet significant ophthalmic features. Additionally, “clinical profile” prompts are incorporated to further improve contextual understanding and diagnostic precision. The proposed method is evaluated through comprehensive performance benchmarking, including quantitative assessments and clinical case studies, to ensure its efficacy in real-world ophthalmic diagnosis.

**Results:**

The experimental findings demonstrate that integrating knowledge graphs and contrastive learning into the MeEYE model significantly improves both diagnostic accuracy and model interpretability. Comparative analyses against baseline models reveal that the proposed approach enhances the identification of ophthalmic conditions with higher precision and clarity. Furthermore, the model’s ability to generate transparent and clinically relevant AI recommendations is substantiated through rigorous evaluation, highlighting its potential for real-world clinical implementation.

**Discussion:**

The results underscore the importance of explainable AI in medical diagnostics, particularly in ophthalmology, where model transparency is critical for clinical acceptance and utility. By incorporating domain-specific knowledge with advanced machine learning techniques, the proposed approach not only enhances model performance but also ensures that AI-generated insights are interpretable and reliable for clinical decision-making. These findings suggest that integrating structured medical knowledge with machine learning frameworks can address key challenges in AI-driven diagnostics, ultimately contributing to improved patient outcomes. Future research should explore the adaptability of this approach across various medical domains to further advance AI-assisted diagnostic systems.

## 1 Introduction

In modern medicine, particularly in ophthalmic diagnosis and treatment, there is an urgent need to develop GPT (Generative Pre-trained Transformer)-based technologies for auxiliary diagnosis (Zandi et al., 2024). The increasing prevalence of eye diseases, which affect visual health and significantly diminish the quality of life, has driven the demand for advanced AI tools like GPT to assist in routine ophthalmic care and diagnosis. Leveraging such technology is very important to address the growing patient load and improve diagnostic efficiency (Tan et al., 2023).

Despite GPT’s impressive natural language processing capabilities, several technical challenges arise when applying it to the specialized field of ophthalmology (Nath et al., 2022). A major issue is the insufficient domain expertise in GPT’s responses. Ophthalmology is a highly specialized discipline that involves complex anatomy, pathophysiology, and diagnostic techniques, all requiring high levels of precision (Wang et al., 2024). Without targeted professional training, GPT-generated responses often fall short of clinical accuracy requirements, potentially leading to unprofessional or misleading information (Biswas, 2023). Additionally, the “black box” nature of computer-aided diagnosis (CAD) models, in which the decision-making process is not transparent, poses a significant barrier to their clinical adoption. Therefore, improving the transparency and interpretability of CAD systems is critical to their integration into clinical practice (Wang et al., 2023).

To address these challenges, this study proposes a novel approach that integrates knowledge graphs (KG) with contrastive learning techniques to enhance patient queries and improve the accuracy and interpretability of GPT-based ophthalmic diagnosis systems (Ni et al., 2024). Knowledge graphs, as structured representations of domain-specific information, effectively capture the intricate relationships between clinical entities and help organize complex medical data (Zhu, 2024). In this study, a comprehensive Ophthalmic Clinical Knowledge Graph (OphKG) was developed through a systematic review of 150 academic papers from the China National Knowledge Infrastructure (CNKI) and Web of Science (WOS) (Wang et al., 2021). OphKG incorporates detailed information such as clinical symptoms, diagnostic markers, treatment outcomes, and patient demographics, serving as a foundation of domain knowledge for AI model training. By integrating this knowledge graph into a contrastive learning framework, the model is better equipped to distinguish subtle differences in clinical presentations, thus enriching the content of patient queries. Contrastive learning improves model performance by identifying differences between similar and dissimilar data, enhancing generalization across various patient groups.

The study integrates patient query graphs with OphKG using contrastive learning (Fang et al., 2023) and introduces a “Clinical Profile”(Younossi et al., 2024) prompt into the patient query process (Benoit, 2023). This prompt, derived from the knowledge graph, includes key clinical attributes such as symptom severity, diagnostic test results, and medical history. The ophthalmology-specific model MeEYE (Wang, 2024), fine-tuned from the CHATGLM3-6B architecture (Yang et al., 2024), was trained using these prompts. In experimental cases, 500 simulated patient queries were conducted to compare the performance of MeEYE with baseline models [ChatGLM3-6B, GPT-4.0 (Taloni et al., 2023), and ERNIE Bot-4.0 API (Zeng et al., 2024)]. The study compared query responses generated with knowledge graph prompts against those from direct queries (the baseline method). The responses were evaluated by three ophthalmology experts, focusing on accuracy, relevance, and interpretability. By incorporating clinical prompts, the model can focus on key clinical features, thus improving both the accuracy and the clarity of the generated diagnostic predictions.

The proposed method aims to enhance the diagnostic performance and transparency of GPT-based CAD systems while providing clinicians and patients with deeper insights into the decision-making process. This is expected to foster greater trust in AI-assisted diagnostics. By integrating domain-specific knowledge graphs and employing contrastive learning and clinical prompt strategies, the method offers a significant advancement in the development of personalized ophthalmic care and diagnostic consultation. The structure of this article includes the methodology in Chapter 2, experimental case results in Chapter 3, a discussion in Chapter 4, and the conclusion in Chapter 5.

## 2 Methodology

**Figure 1:**
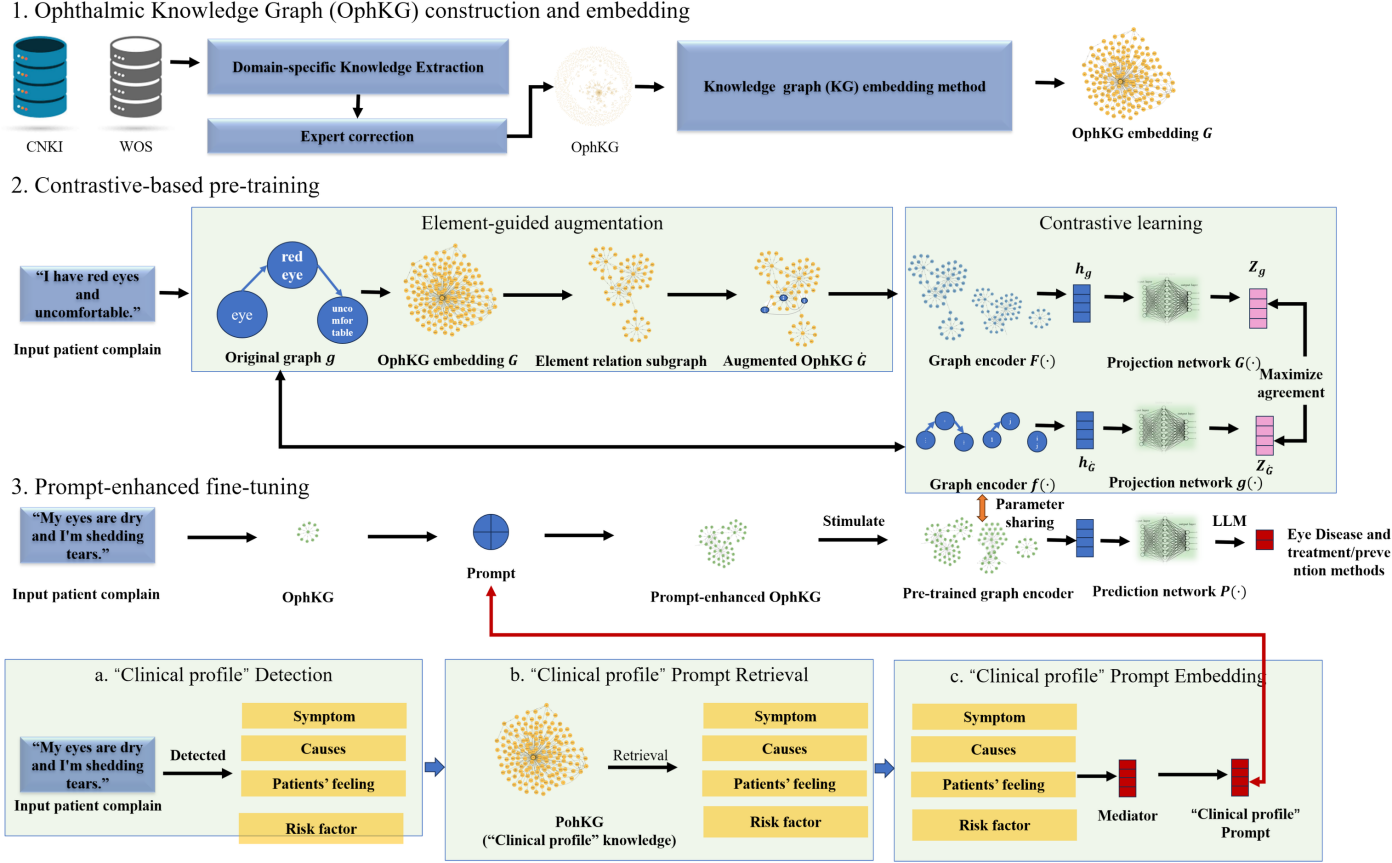
The architecture of knowledge graph-enhanced ophthalmic contrastive learning with ‘clinical profile’ prompt.

As illustrated in Figure 1, this study introduces a prompt framework that leverages a domain-specific knowledge graph and contrastive learning techniques to enhance the accuracy and interpretability of a computer-aided diagnosis (CAD) system. The framework incorporates a “Clinical Profile” prompt to guide the model’s predictions. The proposed methodology is comprised of five key components: the development of an ophthalmic clinical knowledge graph, enhancement of knowledge graph elements through contrastive learning, integration of the “Clinical Profile” prompt, fine-tuning of the large model, and the implementation of a GPT-based ophthalmic auxiliary diagnostic system, followed by a comprehensive evaluation of the method’s effectiveness.

### 2.1 Construction of the ophthalmic knowledge graph (OphKG)

This study constructs an OphKG encompassing key domain-specific knowledge derived from 150 academic papers sourced from CNKI and WOS. The construction process is divided into three primary modules. First, relevant clinical information, including clinical symptoms, diagnostic criteria, treatment interventions, patient demographic data, and clinical outcomes, is extracted from the academic literature. This data is then systematically organized to create a comprehensive knowledge base capturing relationships between ophthalmology-related clinical entities. Second, to ensure the accuracy and reliability of the OphKG, the extracted data undergoes rigorous validation and correction by domain experts. This expert review process is critical for optimizing the knowledge graph, ensuring that it accurately reflects current knowledge and aligns with established ophthalmic clinical guidelines. Lastly, after the knowledge graph is constructed, knowledge graph embedding techniques are employed to convert the structured data into vectorized representations. These embeddings capture the semantic relationships between entities in the knowledge graph, enabling machine learning models to effectively utilize this domain-specific knowledge during the training process.

### 2.2 Contrastive learning and element enhancement

This study utilizes a contrastive learning framework to enhance the original patient question-answer graph, building on the Ophthalmic Knowledge Graph (OphKG). By integrating clinically relevant information, this framework enriches the model’s learning process.

Initially, clinical symptoms, related elements, and relationships are extracted from patient queries to construct the original patient question knowledge graph (denoted as *g*). Using the patient’s initial input, the system employs Jieba for word segmentation and a text classification algorithm (based on dictionaries and BiLSTM+BERT) to classify words into clinical feature categories such as symptoms (observations), causes (underlying reasons), patient experience (subjective feelings), and risk factors (potential contributing factors). These features are identified and linked through the OphKG. For instance, in response to a patient query like “My eyes feel dry and teary, “the model extracts the relevant symptoms, causes, patient experiences, and risk factors from the input data, associating them with the corresponding entities in the knowledge graph.

The original graph g is then enriched by linking it to the corresponding entities and relationships within OphKG, producing an OphKG embedding G . This embedding incorporates additional clinical context and semantic information, enhancing the original patient data with knowledge derived from the graph. By embedding clinical background and domain-specific semantics from OphKG, the patient query data becomes more contextually informed.

Following this, an element-guided graph augmentation technique is applied. An element-relation sub-graph is generated to capture the interrelationships among different clinical features within the original graph. This sub-graph is used to create an augmented graph $\dot{G}$ , which integrates additional connections and clinically relevant information from OphKG, ensuring that the enhanced graph maintains its clinical integrity and relevance.

To extract meaningful representations, independent graph encoders are designed for both the original and enhanced graphs. These encoders generate graph embeddings, which are fed into projection networks to produce the final graph representations. The goal is to maximize the consistency between the original and augmented graph representations while minimizing their similarity to other graphs within the dataset. A contrastive loss function is employed to optimize this process, encouraging the model to produce similar representations for clinically analogous graphs and distinct representations for clinically divergent ones. This approach enhances the model’s ability to generalize across diverse patient profiles and improves its capacity to detect subtle clinical differences in ophthalmic disease presentations.

Finally, the contrastive learning framework strengthens the model’s ability to differentiate between various clinical presentations of eye diseases. Separate graph encoders— $F\left(\cdot \right)$ for the original graph g and $f\left(\cdot \right)$ for the augmented graph $\dot{G}$ , —are employed to extract graph embeddings (denoted as $h_{g}$ and $h_{\dot{G}}$ , respectively). These embeddings are further processed through projection networks $G\left(\cdot \right)$ and $g\left(\cdot \right)$ , yielding final representations $Z_{g}$ and $Z_{\dot{G}}$ . The objective is to maximize the alignment between the representations of the original and augmented graphs while minimizing their similarity to other graph pairs in the dataset. By leveraging a contrastive loss function, the model is optimized to produce congruent representations for clinically similar graphs and distinct representations for clinically dissimilar graphs. This process enhances the model’s ability to generalize across patient profiles and improves its detection of nuanced clinical distinctions in ophthalmic disease.

### 2.3 Integration of the ‘clinical profile’ prompt

The integration of a ‘Clinical Profile’ prompt during the model’s fine-tuning phase enhances its alignment with real-world clinical decision-making processes. Derived from the OphKG, this prompt directs the model’s attention to clinically relevant features, thereby improving the accuracy and contextual relevance of its predictions.

The process begins with the identification of the patient’s clinical profile based on their initial complaint. This clinical profile includes key components such as symptoms (observable signs), causes (underlying mechanisms), patient-reported experiences (subjective symptoms), and risk factors (potential contributors). Using the knowledge graph, the model extracts these elements when processing the patient’s input (e.g., “My eyes are dry and I am experiencing tearing”). At this stage, the model detects the core clinical features associated with the patient’s condition, including symptoms, causes, patient experiences, and risk factors.

In the subsequent stage, the identified clinical profile is utilized to retrieve relevant information from the OphKG. This retrieval process ensures that the model receives a contextually accurate ‘Clinical Profile’ prompt, encapsulating the most critical information about the patient’s condition. The retrieved data refines the model’s focus on the essential clinical features associated with the query, providing a comprehensive understanding of the patient’s symptoms and relevant clinical background.

Finally, the ‘Clinical Profile’ prompt is embedded into the model’s graph representation, a key step in the fine-tuning process. This embedding directs the model’s focus toward the most pertinent clinical features. By integrating this prompt, the model improves both the accuracy and interpretability of its predictions. The prompt acts as a crucial intermediary, guiding the model’s decision-making process and ensuring that its predictions are anchored in clinically significant features.

### 2.4 Prompt-enhanced fine-tuning

The fine-tuning process incorporates ‘Clinical Profile’ prompts, which is exposed to more granular patient data, supplemented by clinical profile prompts derived from the OphKG. These prompts include critical details such as symptoms, causes, patient experiences, and risk factors, all of which are directly relevant to the specific clinical scenario. The inclusion of these ‘Clinical Profile’ prompts is pivotal in the fine-tuning of the CHATGLM3-6B model, a conversational pre-trained model developed by Zhipu AI in collaboration with the Knowledge Engineering Group (KEG) Lab at Tsinghua University. Built on Graph Neural Networks (GNN) and Long Short-Term Memory (LSTM) networks, CHATGLM3 converts input dialogue sequences into graph structures, enhancing its ability to understand and process conversation content. The open-source version, ChatGLM3-6B, retains key advantages of its predecessors, such as smooth conversational performance and ease of deployment.

In this study, the fine-tuned ChatGLM3-6B model forms the basis for developing the ophthalmic large model, MeEYE. This fine-tuning process allows the model to integrate more specific and detailed patient data, in conjunction with clinically relevant feature prompts from the OphKG. These prompts encapsulate key clinical attributes, including symptoms, causes, medical history, and risk factors, closely mirroring real-world clinical contexts. As a result, the model’s internal representation becomes more closely aligned with clinical decision-making processes.

By integrating clinical feature prompts, the model not only leverages the broad features acquired during contrastive learning but also accounts for subtle variations within the clinical context. The contextual information provided by these prompts enables the model to generate more accurate diagnoses. Additionally, the predictions become more interpretable for clinicians, as they are directly linked to specific clinical profiles, ultimately improving both the transparency and interpretability of the diagnostic decision-making process.

### 2.5 Model integration and prediction

To generate accurate and interpretable predictions that comply with established clinical guidelines and expert knowledge, this study compares the MeEYE model with baseline models, including ChatGLM3-6B, GPT-4.0, and ERNIE Bot-4.0 API. Knowledge graph-based prompts were employed to structure and guide the questions posed to each model, supporting predictions related to ophthalmic diseases, as well as their treatment and prevention. This approach ensures that the predictions are both clinically relevant and aligned with expert standards.

### 2.6 Model evaluations

This study rigorously evaluates the performance of the proposed method by comparing it with a baseline model utilizing GPT-4.0 and ERNIE Bot-4.0 LLM APIs. Both models were assessed using a comprehensive dataset of patient inquiries related to ophthalmic diseases, with the baseline model generating predictive responses to the question, “What is the eye disease, and how should it be treated or prevented?” Key evaluation metrics included accuracy, precision, recall, F1 score, AUC-ROC, interpretability score, and diagnostic time. The interpretability score was determined by averaging ratings from three experts based on the model’s outputs.

The proposed method integrates contrastive learning with prompt-based fine-tuning, guided by the OphKG, while the baseline model relies solely on LLM APIs. Predictions from both models were compared against a ground truth dataset validated by clinical experts, and statistical significance tests were performed to identify any significant differences in performance. Additionally, interpretability was assessed through expert reviews and real-world clinical case studies, emphasizing the clarity and clinical relevance of the predictions. This comparative analysis provides a comprehensive understanding of each method’s strengths and limitations, evaluating the proposed approach’s potential to improve diagnostic accuracy, reduce diagnosis time, and enhance the interpretability of AI-driven clinical decision-making.

## 3 Results

### 3.1 OphKG construction

**Figure 2:**
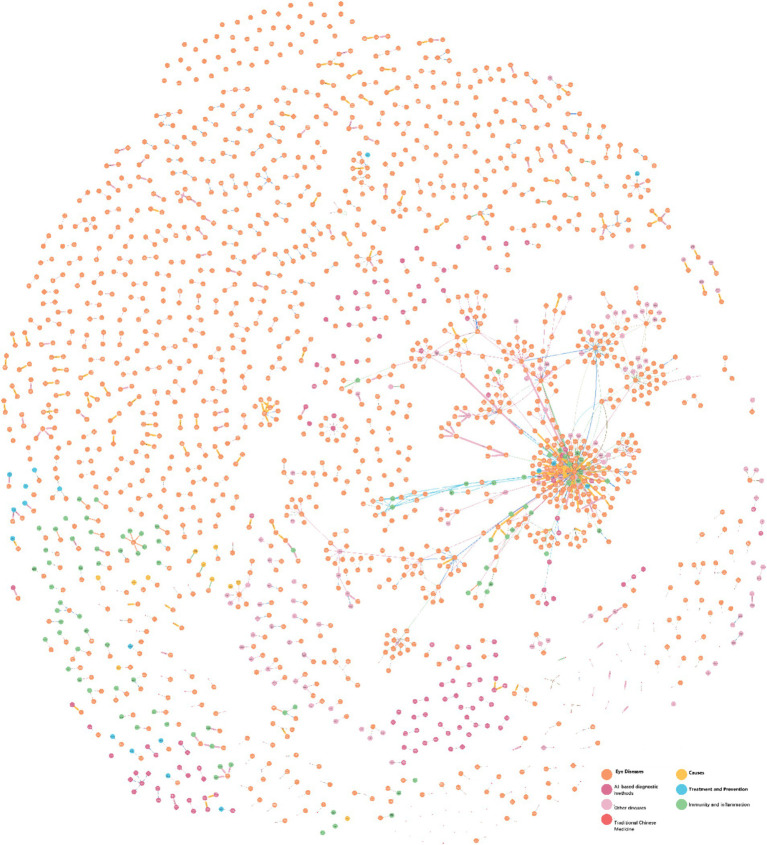
Ophthalmic clinical knowledge graph.

This study developed an Ophthalmic Clinical Feature Knowledge Graph (OphKG) (Figure 2) by extracting three key elements from 150 academic papers in both Chinese and English. The resulting knowledge graph contains 4,125 nodes, 20 types of relationships, and 4,250 attributes, with a peak memory consumption of 1.3 GB. The nodes are categorized into seven distinct clusters: Eye Diseases (orange nodes), Other Diseases (light pink nodes), AI-based Diagnostic Methods (bright pink nodes), Traditional Chinese Medicine (tangerine nodes), Treatment and Prevention (blue nodes), Immunity and Inflammation (green nodes), and Causes (yellow nodes).

### 3.2 Enhanced model performance

This study conducted a comparative evaluation of the proposed MeEYE model by simulating 500 diagnostic questions. The assessment results, as shown in Table 1, indicate that the MeEYE model outperformed baseline models (ChatGLM3-6B, GPT-4.0, and ERNIE Bot-4.0 API) across multiple key performance metrics.

**Table 1:** Model performance evaluation results.

	Metric	Baseline models (ChatGLM3-6B, GPT-4.0, and ERNIE Bot-4.0 API)	MeEYE
“Clinical profile” prompt input	Accuracy (%)	75.3	89.7
	Precision (%)	72.5	88.0.4
	Recall (%)	74.8	90.1
	F1-Score (%)	73.6	89.2
	AUC-ROC	0.82	0.94
	Interpretability score	60	85
	Detection time (seconds)	8	10
Direct question input	Accuracy (%)	74	87.5
	Precision (%)	70.2	86.1
	Recall (%)	73.1	88.2
	F1-Score (%)	71.6	87.1
	AUC-ROC	0.8	0.92
	Interpretability score	58	83
	Detection time (seconds)	7.5	9.5

In the “clinical profile” prompt input, MeEYE achieved an accuracy of 89.7%, significantly higher than the baseline models’ 75.3%. For direct question input, MeEYE’s accuracy was 87.5%, surpassing the baseline models’ 74.0%. These results suggest that MeEYE provides more precise diagnostic outcomes for ophthalmic auxiliary diagnosis tasks. Furthermore, MeEYE demonstrated superior precision, achieving 88.4% for the “clinical profile” prompt and 86.1% for direct questions, compared to the baseline models’ 72.5 and 70.2%. This indicates a lower error rate in predicting positive cases. In terms of recall, MeEYE achieved 90.1% for the “clinical profile” prompt and 88.2% for direct questions, outperforming the baseline models’ 74.8 and 73.1%, demonstrating its enhanced ability to capture true cases with fewer missed diagnoses.

As a comprehensive measure of precision and recall, MeEYE’s F1-Score was 89.2% for the “clinical profile” prompt and 87.1% for direct questions, significantly higher than the baseline models’ 73.6 and 71.6%, further validating its overall performance advantage. MeEYE also achieved AUC-ROC values of 0.94 for the “clinical profile” prompt and 0.92 for direct questions, compared to 0.82 and 0.80 for the baseline models, indicating a substantial improvement in the model’s ability to distinguish between positive and negative cases.

Regarding interpretability, MeEYE scored 85 for the “clinical profile” prompt and 83 for direct questions, markedly higher than the baseline models’ scores of 60 and 58. This demonstrates that the diagnostic outputs of MeEYE are more closely aligned with clinical standards and are easier for clinicians to interpret.

Although MeEYE’s prediction time was slightly longer (10 s for the “clinical profile” prompt and 9.5 s for direct questions), this minor delay is considered acceptable given the significant improvement in diagnostic accuracy and performance.

By integrating “clinical profile” prompts and contrastive learning, MeEYE surpasses traditional baseline models in all ophthalmic auxiliary diagnostic metrics, showcasing its strong potential for practical applications in medical diagnostics.

### 3.3 Case studies

**Figure 3:**
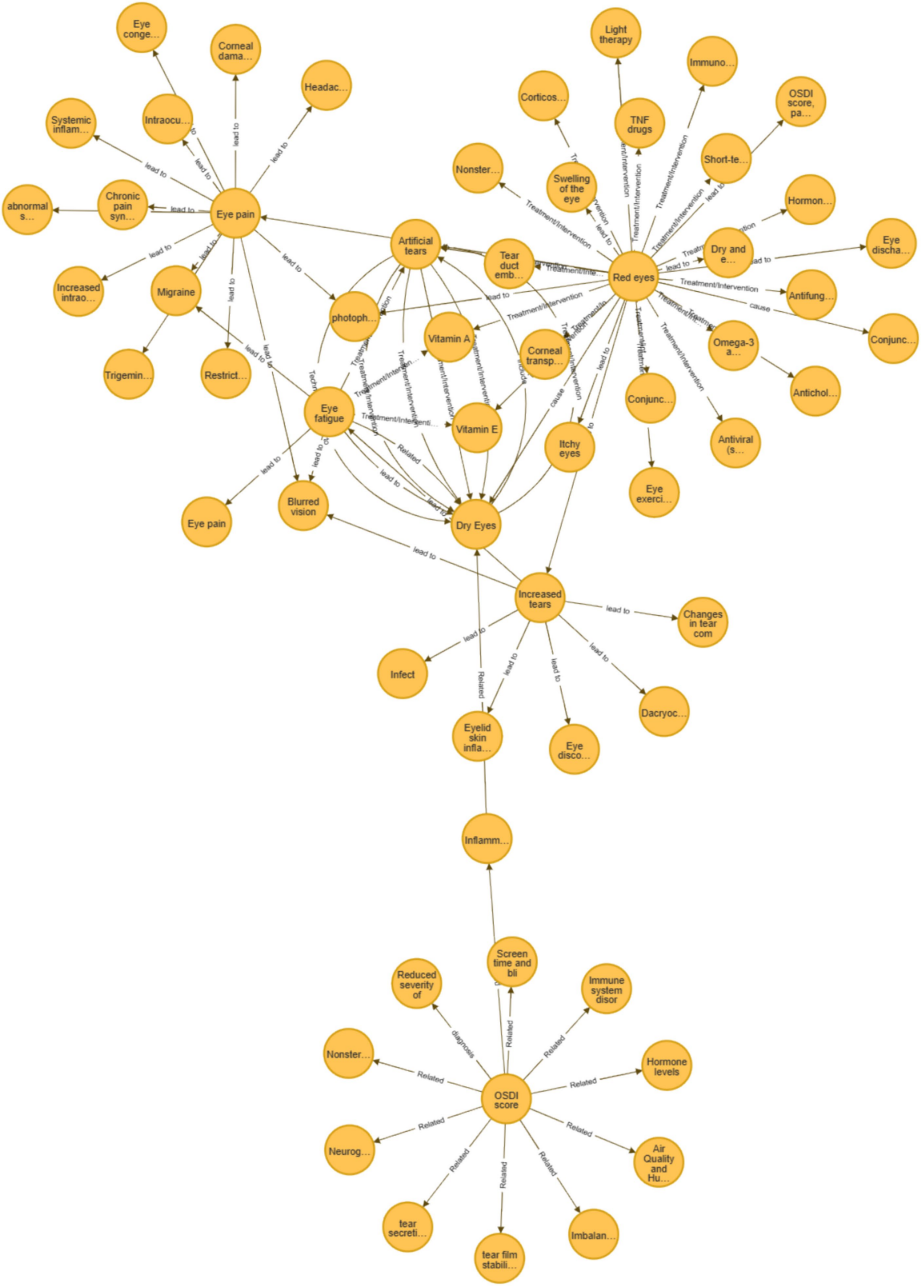
“Red eye” augmentation knowledge graph.

This case study investigates the input “My eyes are red and uncomfortable,” analyzing the diagnostic and treatment recommendations produced by the MeEYE model in comparison to the baseline models (ChatGLM3-6B, GPT-4.0, and ERNIE Bot-4.0 API) using two distinct approaches. Following the application of contrastive learning, an enhanced knowledge graph for “Red Eye” (Wang, 2023) was generated (Figure 3), with Table 2 providing detailed descriptions of the graph’s nodes. The knowledge graph primarily consists of nodes related to the “Red Eye” and incorporates relevant clinical prior knowledge derived from the enhanced graph, facilitating a more informed and comprehensive diagnostic output.

**Table 2:** Retraveled network of “red eye.”

Symptom	Causes	Risk factors	Patents’ feelings
Red eye	Dry eye, conjunctivitis, tear film stability, tear secretion, inflammatory markers (such as IL-6, TNF-α), neurogenic dry eye, air quality and humidity, immune system disorders, hormone levels, microbiome imbalance, screen time and blinking frequency, non-steroidal anti-inflammatory drugs (NSAIDs)	Age ≥ 60, elderly, postmenopausal women	Eye pain, blurred vision, photophobia, eye discharge

The original query in this case study was articulated as: “My eyes are red and uncomfortable.” Utilizing the ophthalmic clinical feature prompt method proposed in this study, the enhanced knowledge graph was transformed into a more detailed representation: “My symptom is red eyes; Possible causes include: dry eye, conjunctivitis, tear film stability, tear secretion, inflammatory markers (e.g., IL-6, TNF-*α*), neurogenic dry eye, air quality and humidity, immune system disorders, hormone levels, microbiome imbalance, screen time and blinking frequency, non-steroidal anti-inflammatory drugs (NSAIDs); I belong to the risk factors of age ≥ 60 years, elderly, postmenopausal women; My symptoms include eye pain, blurred vision, photophobia, eye discharge, discomfort.”

After the user reviewed and refined the input, the final statement was as follows: “My symptom is red eyes; Possible causes include dry eye, conjunctivitis, tear film stability, tear secretion, inflammatory markers, air quality and humidity, screen time and blinking frequency; I belong to the risk factors of age ≥ 60 years, elderly; My symptoms include eye pain, blurred vision, photophobia, eye discharge, discomfort.”

The final query submitted to the GPT model was framed as: “As an ophthalmology expert, please address the following: My symptom is red and swollen eyes; Possible causes include: dry eye, conjunctivitis, tear film stability, tear secretion, inflammatory markers, air quality and humidity, screen time and blinking frequency; I belong to the risk factors of age ≥ 60 years, elderly; My symptoms include eye pain, blurred vision, photophobia, eye discharge, discomfort. What is the most likely ophthalmic disease, and what are the recommended treatments or preventive measures?”

For comparison, the same case was submitted to ChatGLM3-6B, GPT-4.0, and ERNIE Bot-4.0 using a simplified input: “My eyes are red and uncomfortable. What is the most likely ophthalmic disease, and how should it be treated or prevented?”

In the “Red Eye” case, the proposed model demonstrated a markedly superior ability to accurately identify the patient’s symptoms. Using the “clinical profile” prompt input, MeEYE achieved an accuracy of 91.5%, significantly surpassing the baseline models: 74.6% for ChatGLM3-6B, 78.4% for GPT-4.0, and 77.78% for ERNIE Bot-4.0. In the direct question input, MeEYE also achieved a higher accuracy of 89.2%, consistently outperforming the baseline models. This indicates that, when combined with knowledge graph prompts, MeEYE is more effective at addressing complex clinical issues (Table 3).

**Table 3:** Model performance evaluation results of the case.

Question input method	Metric	MeEYE	ChatGLM3-6B baseline	GPT-4.0 baseline	ERNIE Bot-4.0 baseline
“Clinical profile” prompt input	Accuracy (%)	91.5	74.6	78.4	77.78
	Interpretability score	87	60	65	63
	Detection time (seconds)	12	7	8	7
Direct question input	Accuracy (%)	89.2	72.4	76.8	75.9
	Interpretability score	85	58	63	61
	Detection time (seconds)	11	7	8	7

MeEYE’s interpretability score for the “clinical profile” prompt input was 87, far exceeding the scores of ChatGLM3-6B (60), GPT-4.0 (65), and ERNIE Bot-4.0 (63). This reflects MeEYE’s closer alignment with the clinical reasoning process typically employed by physicians, enhancing the transparency and interpretability of its outputs. For the direct question input, MeEYE’s interpretability score was 85, again significantly higher than the other models. Although MeEYE’s diagnosis time was slightly longer at 12 s compared to the baseline models’ 7–8 s, the trade-off is deemed acceptable given the substantial improvements in accuracy and interpretability. For the direct question input, MeEYE’s diagnosis time was 11 s, still longer than the baseline models, but the performance benefits remained evident.

## 4 Discussion

This study enhanced the accuracy and interpretability of ophthalmic disease diagnosis by combining “clinical profile” prompts, contrastive learning, and fine-tuning large language models.

### 4.1 Clinical profile prompts

Clinical profile prompts provide the model with domain-specific contextual information, such as symptoms, potential causes, and patient history. Compared to directly inputting questions, these prompts refine critical details within the query, enhancing the model’s ability to address complex medical cases. By emphasizing specific symptoms or risk factors, the model can more accurately identify and classify conditions, leading to improved diagnostic precision. This approach reduces the likelihood of misdiagnosis that may occur due to the model’s insufficient contextual understanding.

As these clinical prompts are grounded in real-world medical scenarios, the generated predictions are more closely aligned with the clinical reasoning processes used by physicians. The prompts enable the model to focus on clinically significant features, making its outputs more interpretable for healthcare professionals. This is particularly important in the diagnosis of complex ophthalmic conditions, where clinical profile prompts improve the transparency of the decision-making process. As a result, the interpretability of the model’s diagnostic outputs is enhanced, assisting physicians in better understanding and applying the model’s recommendations in clinical practice.

Additionally, clinical prompts allow the model to more effectively manage the variability among individual patients. Since each patient may present with distinct clinical features, prompts enable the model to tailor its diagnostic approach to the specific context of the patient. This targeted prompting enhances the model’s adaptability to diverse cases and minimizes performance degradation when dealing with cross-domain or cross-condition scenarios.

### 4.2 Contrastive learning

The application of contrastive learning significantly enhances the model’s ability to detect clinically relevant distinctions in patient data. Contrastive learning excels at differentiating between similar and dissimilar data pairs, which is particularly advantageous in this study as it enables the model to generalize effectively across diverse patient populations. The OphKG was instrumental in this process by guiding the selection of clinically pertinent data pairs, ensuring that the model’s learning remains closely aligned with established medical knowledge.

A key innovation of this study is the introduction of clinical profile prompts during the fine-tuning phase. These prompts, derived from OphKG, incorporate essential clinical attributes such as symptom severity, diagnostic test results, and patient history. By directing the model’s attention to these clinically significant features, the prompts not only improve the model’s predictive accuracy but also enhance its interpretability. This approach not only boosts diagnostic performance but also provides clinicians with meaningful insights into the AI’s decision-making process, thereby fostering greater trust in AI-driven diagnostic systems.

### 4.3 Fine-tuning large models

Large language models (LLMs), such as ChatGLM3-6B, exhibit strong natural language processing capabilities during the pre-training phase. However, in specialized domains like ophthalmic diagnosis, the performance of general-purpose language models can be limited. Fine-tuning LLMs enables the model to adapt more effectively to the specific datasets and task requirements of the medical field. In this study, the MeEYE model, after undergoing fine-tuning, demonstrated an enhanced ability to manage complex ophthalmic data. When combined with clinical feature prompts from the knowledge graph, it produced more accurate diagnostic outcomes.

The fine-tuned LLM integrates the general language processing capabilities of the base model with specialized medical knowledge. Across various metrics, including accuracy, precision, recall, and F1-Score, the fine-tuned MeEYE model significantly outperformed the baseline models, which had not been fine-tuned. This outcome highlights that fine-tuned LLMs can achieve superior performance in medical diagnostic settings, providing both accurate diagnoses and consistent performance across diverse clinical scenarios.

By undergoing fine-tuning, the model gains the flexibility to handle a range of medical tasks, such as disease classification, symptom analysis, and treatment recommendations. This adaptability makes the fine-tuned model applicable not only to ophthalmic diagnosis but also to other areas of medicine, demonstrating its broad potential for medical applications.

### 4.4 Case study analysis

A case study using the input “My eyes are red and uncomfortable” further validated the effectiveness of the proposed method. By integrating detailed prompts that included specific symptoms, potential causes, risk factors, and patient-reported experiences, the model generated diagnostic outcomes that were both more accurate and clinically relevant compared to the baseline models. In this instance, the proposed method achieved an accuracy of 91.5%, significantly surpassing the baseline models (ChatGLM3-6B at 74.6%, GPT-4.0 at 78.4%, and ERNIE Bot-4.0 at 77.8%). Additionally, the method demonstrated superior interpretability, with a score of 87 compared to 60, 65, and 63 for the baseline models, underscoring its ability to provide clinicians with clear, actionable insights.

Despite the notable advantages demonstrated by the “knowledge graph-enhanced ophthalmic contrastive learning and ‘clinical profile’ prompt” method in diagnosing dry eye, certain limitations remain. First, the model’s performance is highly dependent on the quality and comprehensiveness of the knowledge graph. Since the OphKG was constructed from a limited set of literature, it may not fully capture all clinical variations of dry eye, particularly rare cases or poorly understood pathological mechanisms, potentially constraining the model’s generalizability. Second, while the introduction of clinical profile prompts enhanced interpretability, these prompts are derived from the existing knowledge in the graph and may be inadequate when addressing novel or emerging clinical features. Furthermore, the interpretability score relies on subjective expert evaluations, lacking more objective and quantitative assessment methods.

### 4.5 Future research

Ensuring the interpretability of AI-driven ophthalmic diagnostic models is essential for their clinical adoption and trustworthiness. While this study employs expert evaluations to assess model interpretability, future research should focus on developing a hybrid evaluation framework that integrates both qualitative and quantitative interpretability metrics. Automated techniques such as SHapley Additive exPlanations (SHAP) and Local Interpretable Model-agnostic Explanations (LIME) should be incorporated to provide an objective breakdown of the model’s decision-making process, enhancing transparency and clinical relevance. Additionally, efforts should be directed toward refining expert-based scoring methods by implementing structured assessment rubrics, inter-rater reliability measures, and calibration exercises to ensure consistency across expert evaluations. Longitudinal studies should also be conducted to monitor the evolution of interpretability scores as the model is fine-tuned with real-world clinical data, ensuring that AI-generated explanations remain meaningful and aligned with evolving medical guidelines.

To further enhance the generalizability and real-world applicability of AI-based ophthalmic diagnostic models, future research should prioritize mitigating bias in model training and optimizing computational efficiency. Expanding the dataset to include a more diverse representation of patient demographics, including variations in age, gender, ethnicity, and disease severity, is crucial for improving model fairness and ensuring robust performance across underrepresented subpopulations. Additionally, integrating adaptive learning mechanisms that allow models to update dynamically based on real-world clinical feedback will enhance the ability of AI systems to handle emerging ophthalmic conditions. Optimizing computational efficiency through techniques such as knowledge distillation, model pruning, and edge computing will be necessary to facilitate real-time AI-assisted diagnostics in high-volume clinical settings. These advancements will collectively ensure that AI-driven ophthalmic diagnostic models are not only accurate and interpretable but also scalable, equitable, and practical for widespread clinical implementation.

Thus, future research should prioritize expanding the knowledge graph’s scope, ensuring it encompasses a broader range of ophthalmic conditions and emerging medical knowledge to enhance model generalizability. Additionally, further efforts should focus on developing more adaptable AI models capable of maintaining high diagnostic accuracy across diverse patient populations and complex clinical scenarios. To improve model transparency and clinical trust, the integration of automated and standardized interpretability assessment methods is essential, enabling objective and reproducible evaluations. While this study has demonstrated success in diagnosing dry eye, its applicability to a wider spectrum of ophthalmic diseases and multi-disease diagnostic scenarios remains an open area for investigation. Future studies should validate the model’s performance in diverse real-world clinical environments, assessing its robustness, scalability, and effectiveness in handling complex ophthalmic conditions. These advancements will be critical for ensuring that AI-driven diagnostic tools are not only clinically accurate but also interpretable, equitable, and broadly applicable across ophthalmology and beyond.

## 5 Conclusion

This study presents a novel approach that integrates knowledge graphs with contrastive learning, enhanced by “clinical profile” prompts, to fine-tune the ophthalmology-specific large model, MeEYE, based on the CHATGLM3-6B architecture. This method marks a significant advancement in AI-driven ophthalmic diagnostics, improving both the accuracy and interpretability of diagnostic predictions. By leveraging domain-specific knowledge from the OphKG and employing contrastive learning, the model effectively captures clinically relevant features, offering more reliable and transparent AI-generated recommendations. This addresses the critical need for explainable AI in clinical settings, providing clinicians with clear, trustworthy insights that have the potential to enhance patient care.

The study highlights the successful combination of specialized ophthalmic expertise with advanced AI methodologies, resulting in a system that enhances diagnostic precision and closely aligns with the clinical reasoning used by healthcare professionals. The findings demonstrate that the proposed approach significantly outperforms baseline models in both accuracy and interpretability, emphasizing its ability to manage complex medical cases with greater efficiency. Furthermore, the developed framework, which boosts AI model performance through the integration of knowledge graphs and tailored prompts, has significant potential for application in other medical fields. By enhancing the transparency and reliability of AI-driven diagnostics, this approach could lead to more informed clinical decisions, better patient outcomes, and increased confidence in AI technologies in healthcare.

This research lays the groundwork for future advancements in AI-assisted healthcare. Expanding the knowledge graph to cover a wider range of diseases and incorporating emerging medical knowledge will further enhance the system’s robustness. Additionally, applying this approach to more complex, multi-disease diagnostic scenarios could transform the role of AI in various medical domains. The promising future of this research lies in its potential to revolutionize AI-driven diagnostics, making healthcare more precise, personalized, and widely accessible.

## Data Availability

The datasets presented in this study can be found in online repositories. The names of the repository/repositories and accession number(s) can be found below: https://github.com/MiniHanWang/kg_eye.

## Author’s note

A related Chinese invention patent named “Machine learning based emergency medical prompt method and system for dry eye disease” is applied in August 23, 2024. The application number is 202410685677.
